# DNA-Interactive Properties of Crotamine, a Cell-Penetrating Polypeptide and a Potential Drug Carrier

**DOI:** 10.1371/journal.pone.0048913

**Published:** 2012-11-08

**Authors:** Pei-Chun Chen, Mirian A. F. Hayashi, Eduardo Brandt Oliveira, Richard L. Karpel

**Affiliations:** 1 Department of Chemistry and Biochemistry, University of Maryland Baltimore County (UMBC), Baltimore, Maryland, United States of America; 2 Departamento de Farmacologia, Universidade Federal de São Paulo (UNIFESP), São Paulo, São Paulo, Brazil; 3 Departamento de Bioquímica e Imunologia, Faculdade de Medicina, Universidade de São Paulo (USP), Ribeirão Preto, Brazil; University of Helsinki, Finland

## Abstract

Crotamine, a 42-residue polypeptide derived from the venom of the South American rattlesnake *Crotalus durissus terrificus*, has been shown to be a cell-penetrating protein that targets chromosomes, carries plasmid DNA into cells, and shows specificity for actively proliferating cells. Given this potential role as a nucleic acid-delivery vector, we have studied in detail the binding of crotamine to single- and double-stranded DNAs of different lengths and base compositions over a range of ionic conditions. Agarose gel electrophoresis and ultraviolet spectrophotometry analysis indicate that complexes of crotamine with long-chain DNAs readily aggregate and precipitate at low ionic strength. This aggregation, which may be important for cellular uptake of DNA, becomes less likely with shorter chain length. 25-mer oligonucleotides do not show any evidence of such aggregation, permitting the determination of affinities and size via fluorescence quenching experiments. The polypeptide binds non-cooperatively to DNA, covering about 5 nucleotide residues when it binds to single (ss) or (ds) double stranded molecules. The affinities of the protein for ss- vs. ds-DNA are comparable, and inversely proportional to salt levels. Analysis of the dependence of affinity on [NaCl] indicates that there are a maximum of ∼3 ionic interactions between the protein and DNA, with some of the binding affinity attributable to non-ionic interactions. Inspection of the three-dimensional structure of the protein suggests that residues 31 to 35, Arg-Trp-Arg-Trp-Lys, could serve as a potential DNA-binding site. A hexapeptide containing this sequence displayed a lower DNA binding affinity and salt dependence as compared to the full-length protein, likely indicative of a more suitable 3D structure and the presence of accessory binding sites in the native crotamine. Taken together, the data presented here describing crotamine-DNA interactions may lend support to the design of more effective nucleic acid drug delivery vehicles which take advantage of crotamine as a carrier with specificity for actively proliferating cells.

## Introduction

Crotamine is a 42-residue basic polypeptide derived from the venom of the South American rattlesnake *Crotalus durissus terrificus*. [1] As a highly positively-charged polypeptide, it can function as a cell-penetrating peptide (CPP), crossing the lipid barrier of cellular membranes. [2]–[4] CPPs include the Tat protein from HIV-1, the Antennapedia homeobox protein, and multicationic oligomers such as polyarginines and polylysines. [4]–[8] In addition to traversing the cellular membrane, these peptides can transport proteins, nucleic acids and perhaps, even entire genes across the cellular membrane, [2], [8]–[14] suggesting possible theranostic applications. [15]–[16] An advantageous feature of CPPs in drug transduction is that these carriers do not require specific receptors in the targeted cell membrane.

Crotamine stands out among CPPs for its unique specificity for actively proliferating (AP) cells. [12], [17]. This special property is one of an increasing number of examples of animal toxins with potential therapeutic applications. [8], [12], [17]–[20] Interestingly, crotamine displays antimicrobial activity [21]–[22], and phylogenetic analysis indicates that it shares a common ancestry and a strongly conserved three-dimensional folding pattern with the non-toxic β-defensin antimicrobial peptides, [23]–[24].

Crotamine is non-toxic to mouse stem cells and mammalian cells at low micromolar levels [17], [25] and it can be used to transfect a variety of mammal cells both *in vitro* and *in vivo*. [8], [12], [17], [25] It has been shown to successfully promote the transfection of bone marrow and other AP cells upon intraperitoneal injection of the crotamine-DNA complex. [12] The mechanism by which crotamine or crotamine-DNA complexes enter cells involves binding to cell-surface heparan sulfate proteoglycans, followed by endocytosis. [12] When administered to cells at toxic levels, concentration-dependent intracellular accumulation of crotamine leads to a subsequent permeabilization of endosomal/lysosomal vesicles, and protease leakage that triggers cell death. [25] A recent study demonstrated a selective cytotoxicity of crotamine toward tumor cells: treatment with crotamine showed a significant inhibition of tumor growth and enhanced survival of melanoma tumor-bearing mice. [26] Thus, this polypeptide could serve to target malignant cells *in vivo* with nucleic acids as the direct drug agent, or with intracellularly-expressed gene products encoded by DNA. Recently, selective uptake of crotamine *in vivo* by AP cells in tumor bearing mice models was demonstrated. [27].

The ability of crotamine to permeabilize endosomal/lysosomal vesicles confers an additional and unique advantage to this protein as a CPP drug carrier. The efficiency of cargo delivery by CPPs in the absence of endosomolytic drugs is generally severely limited by their entrapment within endosomes. [14], [28]–[29] Many CPPs also require covalent linkage of the cargo for delivery into cells,[30]–[31] whereas crotamine forms complexes with nucleic acids [12] via ionic forces and other non-covalent interactions, as will be detailed in the present work.

Formation of crotamine-DNA complexes was initially indicated by alterations in the CD spectra of protein-plasmid DNA mixtures compared to the spectra of each molecule separately, and also by the loss of DNA electrophoretic mobility in the presence of crotamine. [12] Given the potential of crotamine as a DNA-delivery vehicle with additional anti-cancer and anti-microbial activities, [21] and its ability to bind to the chromosomal DNA of mammalian cells after internalization, [12], [17] the study of crotamine interaction with nucleic acids is of major importance. The utility of crotamine as a nucleic acid-delivery vehicle will likely depend significantly on the affinity of crotamine for DNA, and the factors that influence this affinity. As pointed out by Ziegler and Selig, the CPP-DNA binding affinity must be high enough to stabilize the resulting complex and promote high transfection, but it also needs to be sufficiently low in order to facilitate the release of the DNA cargo upon cellular uptake. [13] We further note that the demonstrated affinity of crotamine for chromosomal DNA [17] might play an essential role for efficient delivery of therapeutic genes into the cell nucleus mediated by this protein.

Herein we describe in detail the binding of crotamine to single- (ss) and double-stranded (ds) DNAs of different lengths, base compositions, and structure over a range of ionic conditions. Agarose gel electrophoresis and ultraviolet spectrophotometry analysis indicate that crotamine complexes with long-chain DNAs readily aggregate and precipitate at low ionic strength. This aggregation becomes less likely with shorter chain length or increasing ionic strength. The ss- or ds-oligonucleotides containing, respectively, 25 nucleotides or base pairs did not show any evidence of such aggregation, thus permitting determination of affinities and site size via fluorescence quenching experiments. We estimate that when it binds to ssDNA, crotamine occludes about 5 nucleotide residues, and about the same number of residues (∼2.5 base pairs) upon interacting with dsDNA. The affinities of the protein for ss- vs. ds-DNA were comparable and, as expected, decreased with increasing salt levels. Analysis of the dependence of the affinity on [NaCl] indicates a maximum of ∼3 ionic interactions between the protein and DNA. A short peptide, Arg-Trp-Arg-Trp-Lys-Leu-NH_2_, containing residues 31–35 of crotamine, corresponding to a potential DNA binding site, bound DNA with a lower affinity and salt dependence than that of the full protein. This suggests that crotamine could possess more than one DNA binding site.

The development of crotamine as an effective drug carrier will require detailed knowledge of its DNA binding properties, and the results of this should prove most valuable in this process.

## Materials and Methods

### Materials

Crotamine was purified from *C. durissus terrificus* venom as previously described. [17] The venom was obtained as a gift from the Faculdade de Medicina de Ribeirão Preto (FMRP) serpentarium, São Paulo University. Stock solutions of lyophilized protein in the standard buffer (see below) were kept frozen at −20°C until use. Protein concentration was determined spectrophotometrically at 280 nm using the extinction coefficients and composition of the component aromatic amino acids, Trp, Tyr, and Phe, respectively: ε_280_ = 2×5559+1197+0.7 = 1.23×10^4^ M^−1^ cm^−1^. Oligonucleotides were obtained from Midland Certified Reagent Company, Inc. (Midland, TX): KR-2: d(CCG)_8_C, KR-1: dG(CGG)_8_, 21+: d(ATGTGGAAAATCTCTAGCAGT), 21-: d(ACTGCTAGAGATTTTCCACAT), (dT)_7_, (dT)_14_, and (dT)_21_. Calf thymus DNA was purchased from Sigma (type I, estimated by the supplier to be 10–15 MDa in length).

The synthesis of the Arg-Trp-Arg-Trp-Lys-Leu-NH_2_ was carried out manually using standard Fmoc (N-(9-fluorenyl)methoxycarbonyl) chemistry [32] on 100 mg of Rink amide resin (0.7 meq/g; Advanced Chemtech, Louisville, KY, USA). Fmoc-Leu-OH, Fmoc-Lys(Boc)-OH, Fmoc-Trp(Boc)-OH and Fmoc-Arg(Pbf)-OH (Advanced Chemtech) were used in 2.5-fold molar excess relative to the nominal resin derivatization. Couplings were performed by use of HBTU (O-benzotriazole-N,N,N',N'-tetramethyl-uronium hexafluoro-phosphate)/HObt (N-hydroxybenzotriazole monohydrate) mixture in presence of N,N'-diisopropylethylamine. Removal of side chain protection and cleavage of the peptide from the Rink amide resin were simultaneously performed by using 2×2 mL trifluoroacetic acid, water, triisopropylsilane, anisol (88∶5:4∶3 v/v/v/v) plus 20 mg dithiotreitol for 2 h at room temperature. The crude peptide solution thus obtained was concentrated under vacuum to an oily residue which was then extracted four times with 5 mL of cold t-butyl methyl ether to remove reaction by-products. Further purification was performed by applying the crude peptide dissolved in 10 mL of 0.15 M NaCl solution buffered with 0.03 M Tris-Cl, pH 8.1, into a 1×40 cm column of CM-Sepharose FF (Amersham Biosciences) equilibrated with sample buffer and developed with a NaCl gradient up to 1 M. The material corresponding to the latest eluting A_280_ nm peak was characterized by amino acid analysis after acid hydrolysis, [33] indicating a peptide whose composition had the molar ratio Leu (1.0), Lys (1.2), Trp (2.0) and Arg (1.8) and an overall yield of 22%. Aliquots of purified peptide were desalted by reverse phase chromatography in C-18 Sep-Pak cartridges (Millipore Co., MA, USA) and lyophilized.

Unless otherwise stated, the standard buffer used for protein-DNA binding and aggregation experiments was 0.02 M Hepes, pH 7.7, 0.0001 M Na_2_EDTA, and varying levels of NaCl.

### Electrophoresis

Electrophoresis experiments utilized TopVision LE GQ Agarose (low electroendosmosis, Fermentas Life Sciences). Unless otherwise indicated, gels contained 3% (w/v) agarose and were run in TBE buffer. DNA ladders were obtained from Fermentas Life Sciences (GeneRuler Low Range, 25–700 bp).

### Fluorescence and UV-Visible Spectrometry

Fluorescence titrations were conducted in a SPEX Fluoromax-2 spectrofluorimeter at 20±1°C, with excitation set at 295 nm (2 nm dispersion) and emission at 353 nm (4 nm dispersion). Small (typically 10 µL) aliquots of nucleic acid were added to 2.5 mL of crotamine (1.5 µM) in the standard buffer plus [NaCl], where indicated. After each addition, the solution was stirred for 20 sec. In order to account for any loss of fluorescence due to adhesion of protein to cuvette surfaces, control titrations were performed where aliquots of the standard buffer were added in the same volumes as the nucleic acid aliquots, with subsequent stirring. The displayed fluorescence titrations were adjusted for background (buffer alone) readings, volume changes, and any changes in fluorescence seen with the control experiments. All fluorescence experiments were performed under conditions where absorbance measurements (performed with 50–100 fold greater concentrations of DNA and crotamine) indicated that no aggregation would occur during the course of the titration.

For absorbance measurements, a Cary 100 Bio UV-visible spectrophotometer was used with 200 µL samples prepared in the standard buffer at room temperature.

### Binding Analysis

Fluorescence quenching experiments were analyzed via the McGhee-von Hippel non-specific binding model [34] as modified by Tsodikov *et al* [35] for finite lattices (in this case, oligonucleotides). A non-linear least squares fitting program (NFIT) was used to determine association constants and binding site size. Use of the finite lattice model yielded calculated values for association constant, K, that were slightly (∼15%) higher than those values obtained without the Tsodikov *et *al modification.

## Results and Discussion

### Aggregation of Crotamine-DNA Complexes: Effect of DNA Length and Concentration

**Figure 1:**
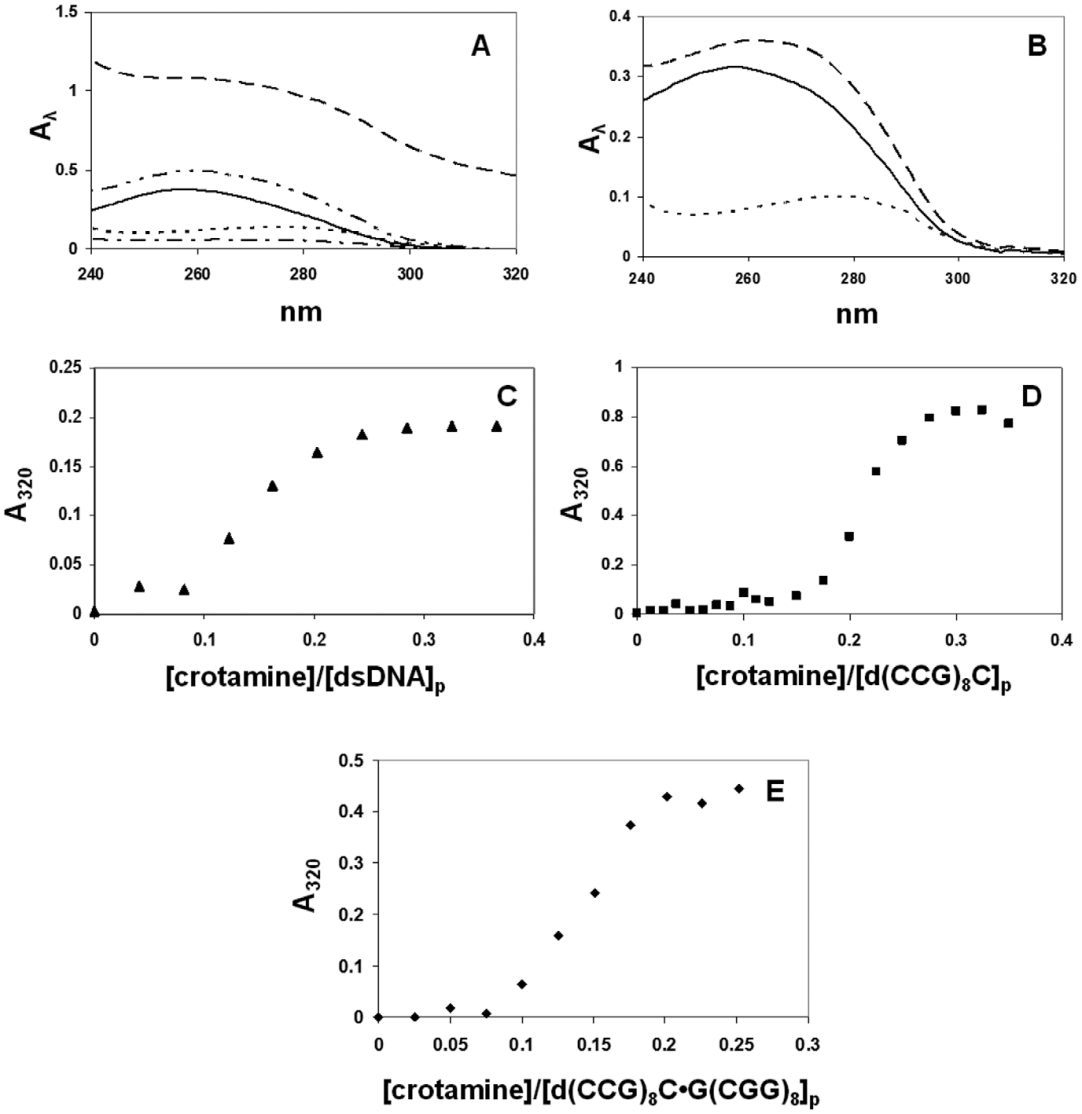
Spectrophotometry of crotamine-DNA mixtures in 0.02 M Hepes, pH 7.5, 0.01 M NaCl, 0.0001 M EDTA (standard buffer with indicated [NaCl]). A. Spectra of 4.1×10^−5^ M ds calf thymus DNA +4.3×10^−6^ M crotamine. ––––––, DNA alone; · · · · ·, crotamine alone; – – – –, DNA + crotamine prior to centrifuging; – · – · – · –, DNA + crotamine supernatant after centrifuging; – · · – · · – · · –, sum of individual DNA and crotamine spectra. B. Spectra of 2.5×10^−5^ M(p) 25-mer (CCG)_8_C and 2.5×10^−6^ M crotamine. ––––––, DNA alone; · · · · ·, crotamine alone; – – – –, DNA + crotamine. C. Titration of ds calf thymus DNA with crotamine; starting [DNA] = 2.22×10^−5^ M(p). D. Titration of d(CCG)_8_C with crotamine; starting [DNA] = 1.21×10^−4^ M(p). E. Titration of d(CCG)_8_C•G(CGG)_8_ with crotamine; starting [DNA] = 6.2×10^−5^ M(p).

Previous results indicated that crotamine readily forms a complex with plasmid DNA, as evidenced by the severely limited electrophoretic mobility, or disappearance of the ethidium bromide-stained DNA in the mixture. [12] In order to determine the extent of aggregation in these complexes, we have examined the spectrophotometric and electrophoretic properties of crotamine-DNA mixtures. As seen in Figure 1A, mixtures of crotamine with double-stranded (ds) calf thymus DNA yielded absorbance readings significantly greater than the sum of the individual protein and nucleic acid absorbances, with light scattering seen at 320 nm. This suggests that aggregation was occurring. Accordingly, when the mixture was spun in a microfuge for 5 min at 13,200 rpm, the supernatant had lost ∼85% of its initial absorbance at 260 and 280 nm, and no absorbance was observed at 320 nm (Fig. 1A). An analogous experiment was performed with a 25-residue single-stranded (ss) oligodeoxynucleotide, d(CCG)_8_C. At comparable concentrations (about 25 µM DNA in nucleotide residues, and 2.5 µM crotamine), no increase at 320 nm occurs when crotamine is mixed with this oligonucleotide (Fig. 1B).

The observed aggregation may be related to the neutralization of positive charge on the protein by the negatively-charged nucleic acid. To test this, we monitored the effect of increasing levels of crotamine on the A_320_ readings seen with calf thymus DNA and with ss- and ds-oligonucleotides. With relatively low levels of ds calf thymus DNA (16.5 µM residue), 320 nm light scattering was observed when the [crotamine]:[DNA residue] exceeded 1∶10 (Fig. 1C). At this DNA concentration, the d(CCG)_8_C showed little evidence of scattering when crotamine was added. At a higher concentration (121 µM residue), mixtures of the 25-mer oligonucleotide and crotamine displayed a more abrupt increase in scattering, occurring at a [crotamine]:[DNA residue] of ∼1∶5 (Fig. 1D). Note that the spectrum of the oligo-crotamine mixture shown in Fig. 1B was obtained at a significantly lower DNA level and at a [protein]:[DNA residue] just below the point in the titration where the A_320_ sharply increases. The double-helix of the 25-mers and its complement also showed an abrupt transition, which occured at a [crotamine]:[DNA residue] similar to that seen with ds calf thymus DNA (Fig. 1E).

**Figure 2:**
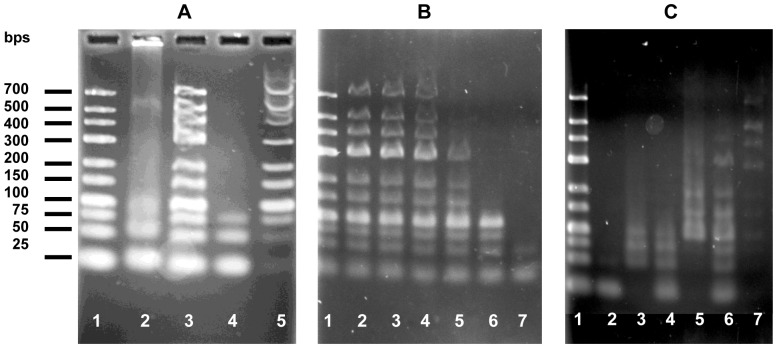
DNA length, concentration, and salt dependencies of crotamine-DNA aggregations, in the standard buffer, unless otherwise indicated. A. Effect of DNA length. Lane 1: DNA ladder; lane 2∶1 µg (3 nmol in residue) DNA ladder +0.3 nmol crotamine, [crotamine]:[DNA]_p_ = 1∶10; lane 3: same as lane 2, but treated with SDS (0.1% net) prior to electrophoresis; lane 4: supernatant of mixture as in lane 2 after centrifugation at 13,000 rpm for 5 min; lane 5: precipitate of mixture in lane 4 after treatment with 0.1% SDS. B. Effect of relative DNA concentration. Each lane contained 0.5 µg DNA ladder. Samples were centrifuged at 13,000 rpm for 5 min, and supernatants applied to the gel. Lane 1: DNA alone. [crotamine]:[DNA]_p_ = 1∶30 (lane 2), = 1∶20 (lane 3), = 1∶15 (lane 4), = 1∶12 (lane 5), = 1∶10 (lane 6), = 1∶7.5 (lane 7). C. Effect of salt. Each lane contained 1 µg DNA ladder. Lane 1: DNA ladder alone. Lanes 2–7∶0.5 µg DNA ladder +0.3 nmol crotamine in 0.01 M NaCl (lane 2: supernatant, lane 3: SDS-treated precipitate), in 0.05 M NaCl (lane 4: supernatant, lane 5: SDS-treated precipitate), in 0.1 M NaCl (lane 6: supernatant, lane 7: SDS-treated precipitate).

These results suggest that aggregation is dependent on both DNA length and concentration. In order to directly assess the effect of DNA length on aggregation, we utilized agarose electrophoresis to determine the solubility of mixtures of crotamine with a dsDNA ladder with lengths varying between 25 and 700 base-pairs (bps). In these experiments, the DNA residue concentrations were considerably higher (250 to 500 µM residue) than the levels used in the spectrophotometric studies, thus increasing the probability of aggregation (UV spectra of these mixtures displayed scattering at 320 nm, although less than that observed with calf thymus DNA; not shown). When a mixture of crotamine and DNA ladder ([protein]:[DNA residue] = 1∶10) was run on 3% agarose, only the shortest DNA chains (25, 50, and 75 bps) were seen to enter the gel (Fig. 2A, lane 2). The longer DNA chains largely remained in the sample well, indicating that they were part of a large protein-DNA aggregate (lane 2). When treated with 0.1% SDS, all the bands were seen to migrate to their expected positions, indicating that the aggregate had been effectively broken up by the detergent (lane 3). When a 1∶10 [protein]:[DNA]_p_ mixture was subjected to a five minute microfuge spin (the “p” refers to nucleotide residue concentration), the supernatant clearly contained the 25, 50 and 75 bp bands (lane 4), which were depleted from the SDS-treated precipitate (lane 5).

The presence of increasing amounts of the 50 and 75 bp chains in the precipitate is in line with the DNA length dependence of aggregation suggested by the spectrophotometric results. This was further illustrated by observing the effect of increasing levels of protein on the electrophoretic mobility of the DNA bands (Fig. 2B). As the [crotamine]:[DNA] increases, the longest DNA strands are the first to be removed from the body of the gel. At the highest [crotamine]:[DNA]_p_ (1∶7.5), only the shortest chains (25, 50, 75, and 100 bps) are visible in the gel. The loss of electrophoretically mobile DNA bands is clearly a function of ionic strength, since increasing levels of NaCl were seen to increase the amount and extent of DNA entering the gel (Fig. 2C). Thus, at 0.10 M NaCl, bands as long as 400 bp are seen in the supernatant of centrifuged crotamine-DNA mixtures. As in Fig. 2A, the electrophoretic patterns of the SDS-treated precipitates show the bands depleted from the supernatants. When the sample wells were located in the middle of the gel, no ethidium bromide-stainable material was seen to migrate towards the negative electrode (data not shown). Thus, there was no evidence for any positively-charged electrophoretic species.

The spectrophotometric and electrophoretic results indicate that crotamine readily forms precipitable aggregates with both ds and ss DNA. The probability of aggregation increases with increasing DNA length, and decreases with increasing ionic strength. Aggregation is clearly dependent on the [protein]:[DNA residue] ratio, and the point where this becomes apparent is likely related to the neutralization of the nucleic acid’s negative charge by the protein. This is a qualitative measure of the occluded site size in the complex, i.e., the number of nucleotide residues covered by each bound protein.

### Binding Site Size and Affinities of Crotamine for Oligonucleotides

In order to directly quantify binding parameters, including affinities, site size and salt dependence, it is necessary to perform experiments under conditions where aggregation does not occur. Thus, these studies are necessarily restricted to relatively short DNA molecules where there is no absorbance at 320 nm upon interaction with the protein. We find that the intrinsic tryptophan (Trp) fluorescence of crotamine is quenched upon interaction with ss and ds oligonucleotides. Fluorescence measurements require only micromolar levels of crotamine, conditions where interacting oligonucleotides do not form aggregates. The binding parameters obtained in these experiments, crotamine-DNA association constants, site sizes, and number of charge interactions associated with binding apply also to longer DNA molecules. However, crotamine molecules interacting with the longer DNAs may also be capable of binding more than one of these substrates (see below).

**Figure 3:**
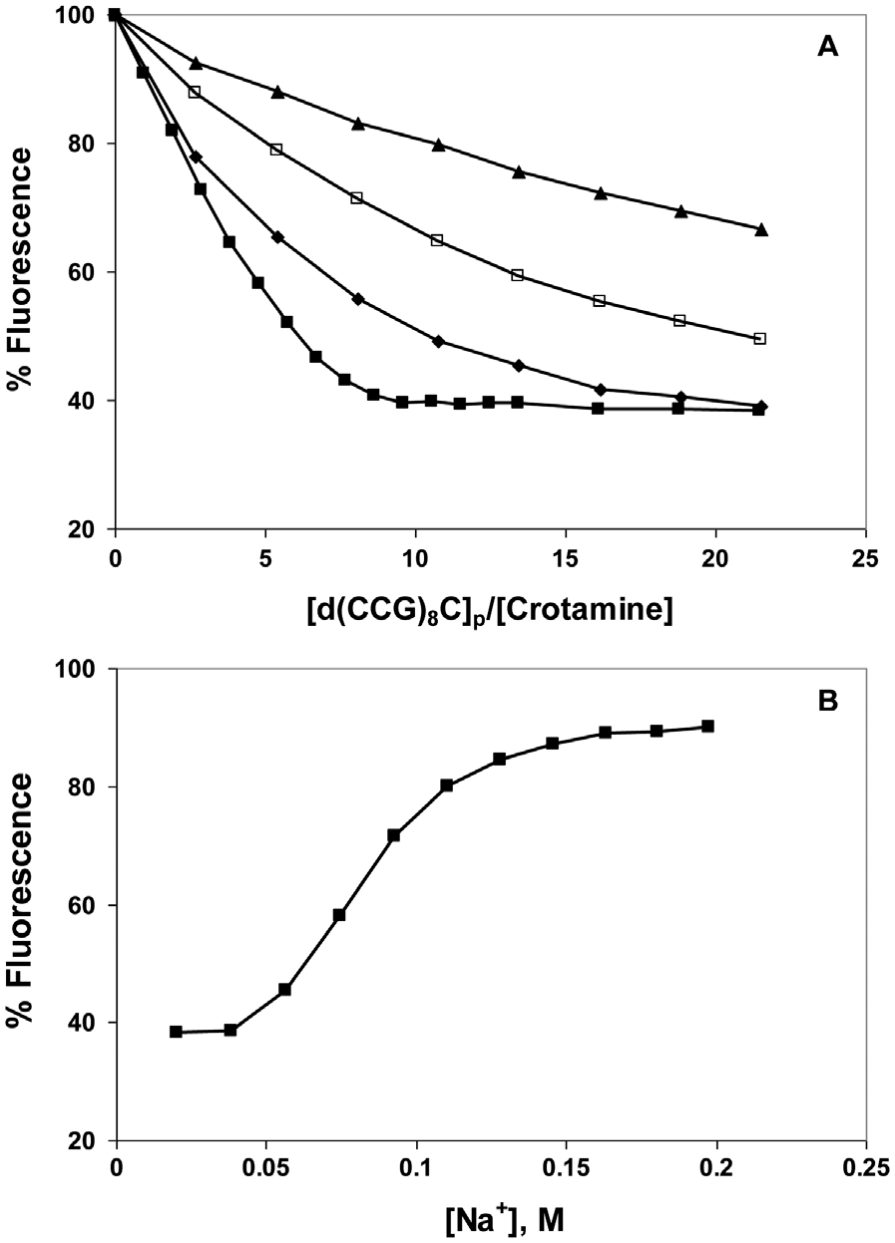
Binding of d(CCG)_8_C to crotamine as a function of [NaCl]. A. Fluorescence titrations were performed in the standard buffer (0.02 M Hepes, pH 7.5, 0.0001 M EDTA) with the following [NaCl]: ▪, 0.01 M; ♦, 0.05 M; □, 0.075 M; ▴, 0.1 M. B. Reversal of 0.01 M titration by addition of aliquots of a concentrated solution of NaCl. [Na+] was calculated from the [NaCl] and the contribution of the other components of the buffer. The lines connect the points and are shown for clarity.

A typical set of titrations, with the ss 25-mers, d(CCG)_8_C, is shown in Fig. 3A. At the lowest salt level, 0.01 M NaCl, the fluorescence drops nearly linearly with the addition of the DNA, reaching a maximal quenching of about 60%. This suggests that under these ionic conditions the binding is close to stoichiometric, *i.e.*, below the saturation point, nearly all the DNA added binds to the protein, with only a small amount free in solution. Upon saturation, the fluorescence no longer drops, so the overall binding plot approximates two straight lines. The intersection of these lines is a measure of the occluded binding site size, although it is not a precise determination. When the experiment is repeated at higher [NaCl], the plots are shallower and clearly non-linear, indicating that not all the added DNA binds protein (Fig. 3A). The addition of aliquots of concentrated NaCl upon completion of the titration restores most of the initial crotamine fluorescence (Fig. 3B). Under the conditions of these experiments, 0.08±0.01 M [NaCl] is needed to restore half the initial fluorescence, and therefore dissociate 50% of the complexes that had been formed.

**Figure 4:**
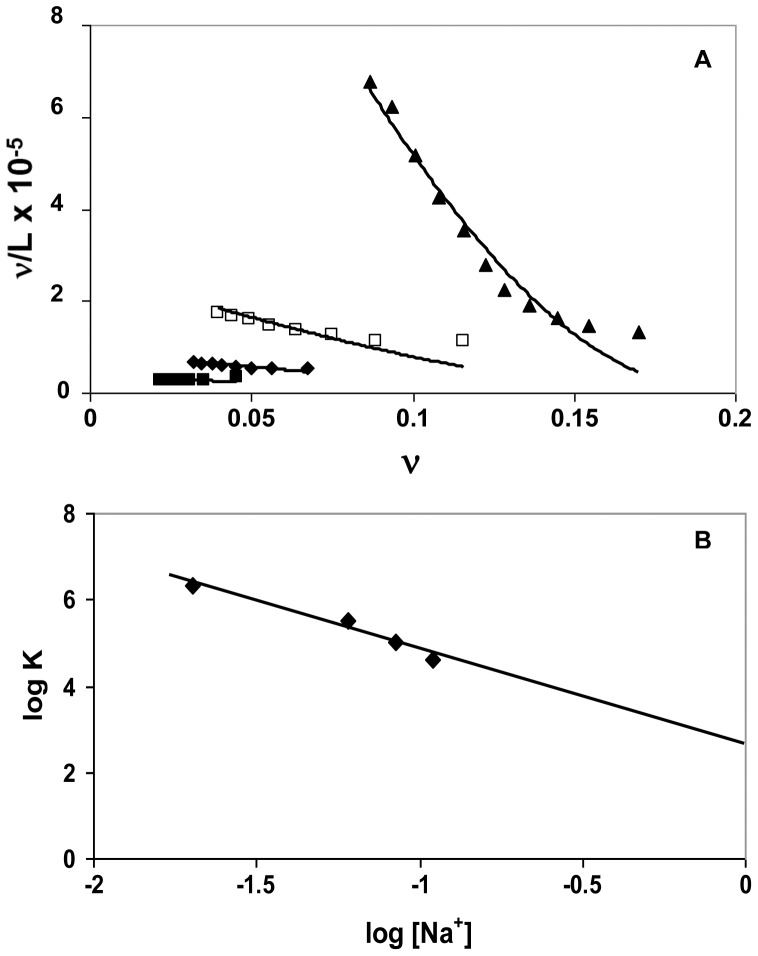
Scatchard plots and salt dependence of the data in Fig. 3. A. Scatchard plot: ν ≡ [crotamine]_bound_/[total d(CCG)_8_C]_p_, L ≡ [crotamine]_free_. The solid lines represent curves calculated from the best fit of the Tsodikov et al. [35] modification of the McGhee-von Hippel model for non-cooperative binding ligands [34] (see Materials and Methods). B. Log-log dependence of the association constants calculated in panel A on [Na^+^].

The data in Fig. 3A was analyzed using the McGhee-von Hippel model for a ligand (protein) binding in a non-cooperative fashion to a lattice (DNA), which accounts for the presence of overlapping binding sites (Fig 4A). [34], [36] The occluded site size, *n*, was found to be 4.6±0.2 nucleotide residues per crotamine; the protein-DNA association constants, *K* (the affinity of the protein for an isolated site on the DNA), are listed in Table 1. At saturation, crotamine-DNA complexes would in principle be positively-charged, since at neutral pH each protein carries a net charge of 8+, and the ∼5-residue DNA segment to which it is bound has a charge of 5-. As we noted above, electrophoresis experiments showed no evidence for positively-charged complexes. In all likelihood, under the conditions of those experiments (which were conducted at crotamine concentrations more than 100-fold higher than the micromolar levels used in the fluorescence studies), aggregation occurred before saturation could be achieved. Furthermore, if each crotamine is capable of binding more than one DNA molecule, which might occur with longer DNAs (see below), the net charge associated with each bound crotamine could be negative, given the additional negative charge provided by the second bound nucleic acid.

**Table 1:** Dependence of Crotamine - d(CCG)_8_C Association Constants on [NaCl]*.

[NaCl], M	[Na^+^], M	*K,* M^−1^
0.010	0.020	2.1±0.2×10^6^
0.050	0.060	3.1±0.3×10^5^
0.075	0.085	1.1±0.1×10^5^
0.100	0.110	4.3±0.4×10^4^

* Titrations were performed in 0.02 M Hepes, pH. 7.7, 0.0001 M Na_2_EDTA, and the indicated concentration of NaCl. The [Na^+^] includes the contribution from the buffer (0.01 M).

**Figure 5:**
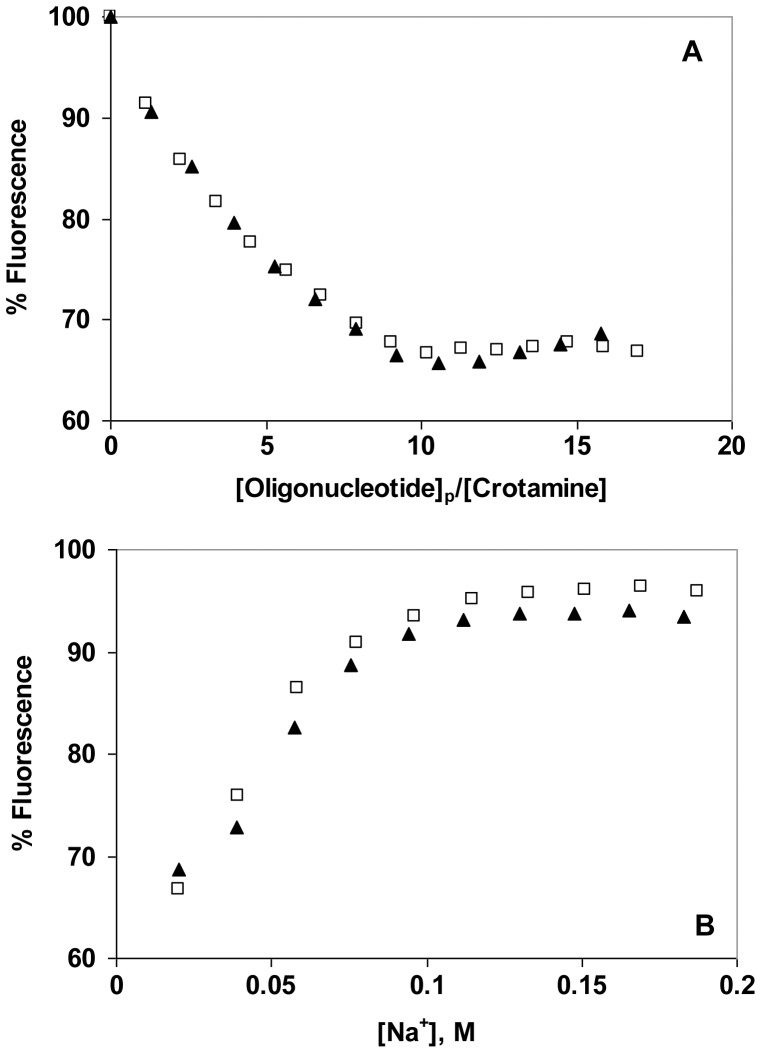
Comparison of binding of a single-stranded oligonucleotide (d(CCG)_8_C) to crotamine with the binding of a double-stranded oligonucleotide (d(CCG)_8_C•G(CGG)_8_). A. Fluorescence titration in the standard buffer with 0.01 M NaCl: □, d(CCG)_8_C); ▴, d(CCG)_8_C•G(CGG)_8_. B. NaCl-induced reversal.

The dependence of *K* on [Na^+^] follows a linear log-log relationship, with a slope of −2.2±0.3 (Fig. 4B). Record and colleagues have shown that the number of ion pairs, *i.e.*, charge interactions, involved in the binding of a protein ligand to a polynucleotide lattice can be calculated from the slope of this log-log dependence:[37]–[38].
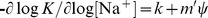



where *k* is the number of anions (in this case Cl^-^) displaced from the protein upon binding, *m′* is the number of cations (in this case Na^+^) displaced from the nucleic acid, and *ψ* is the fraction of counterion bound in the thermodynamic sense per lattice charge (which is generally 0.7–0.8; *i.e.*, on average, 70–80% of phosphates are bound to cations in the free DNA). [37] Thus, depending on the extent of Cl^-^ binding, there are as many as 3 ion pairing interactions in the crotamine-DNA complex. The extrapolated value of *K* at 1 M Na^+^, 440 M^−1^, is the non-ionic component of the affinity. [37] We note that the data points in Fig. 5 are necessarily far from 1 M Na^+^, which adds to the error in the quantitation of this value (with the standard deviation of the extrapolated log *K* of ±0.34, the extrapolated values of *K* fall within a range of 200 to 950 M^−1^). Moreover, this estimate is based on the linearity of the log *K vs.* log [Na^+^] plot (Fig. 4B). It has been observed that the dependence can change at very low ionic strength, producing curvature in the plot. [39] If the data obtained at the lowest [Na^+^] (0.02 M) are excluded, the slope decreases to −3.3±0.2, which could represent as many as ∼4 ion pairs in the protein-DNA complex, still consistent with the occluded site determination. The extrapolated value of *K* at 1 M Na^+^, where the affinity is the result of non-ionic interactions, is 32±12 M^−1^; a ΔG of ∼ −2 kcal mol^−1^. This is lower than the value obtained with all the data points (440 M^−1^, ΔG ∼ −4 kcal mol^−1^), but does not change our observation that at least some of the binding is associated with non-charged residues. Interestingly, it was estimated that the non-ionic contribution to the interaction of crotamine with the negatively-charged polysaccharide, heparin, is also significant: *K* at 1 M Na^+^ is about 1700 M^−1^. [12].

### Effect of DNA Structure and Length on Affinity for Crotamine

We have presented data showing that both ds and ss DNA interact with crotamine. In order to determine if there is any preference for ds *vs.* ssDNA, we compared the binding of ss- and ds-oligonucleotides. The effect of d(CCG)_8_C• dG(CGG)_8_ on crotamine fluorescence in 0.01 M NaCl is shown in Fig. 5, along with the NaCl-induced “salt-back”. The analogous data for dG(CGG)_8_ is plotted on the same graphs. The two sets of data are virtually identical: the maximal extent of quenching is ∼30%, and the complex formed is 50% dissociated at 0.05±0.01 M Na^+^. The near identity of the titrations indicates that the number of nucleotide residues covered by each crotamine on dsDNA is the same as on ssDNA. Thus, crotamine occupies a span of about two and a half nucleotide pairs, so that about four crotamine molecules bind within a 10 bp turn of a B-DNA helix. For either ss or dsDNA, the occluded site size is consistent with the estimate of ≤3 ion pairs between each bound crotamine and the DNA backbone. Note that the half-way point of the d(CCG)_8_C salt-back was reached at 0.08 M Na^+^, and the extent of quenching was twice what was observed with its complement and the duplex formed with its complement. These results do not indicate a clear preference for ss *vs.* dsDNA, although sequence and/or base composition might influence binding affinity, and clearly affect the extent of fluorescence quenching.

**Figure 6:**
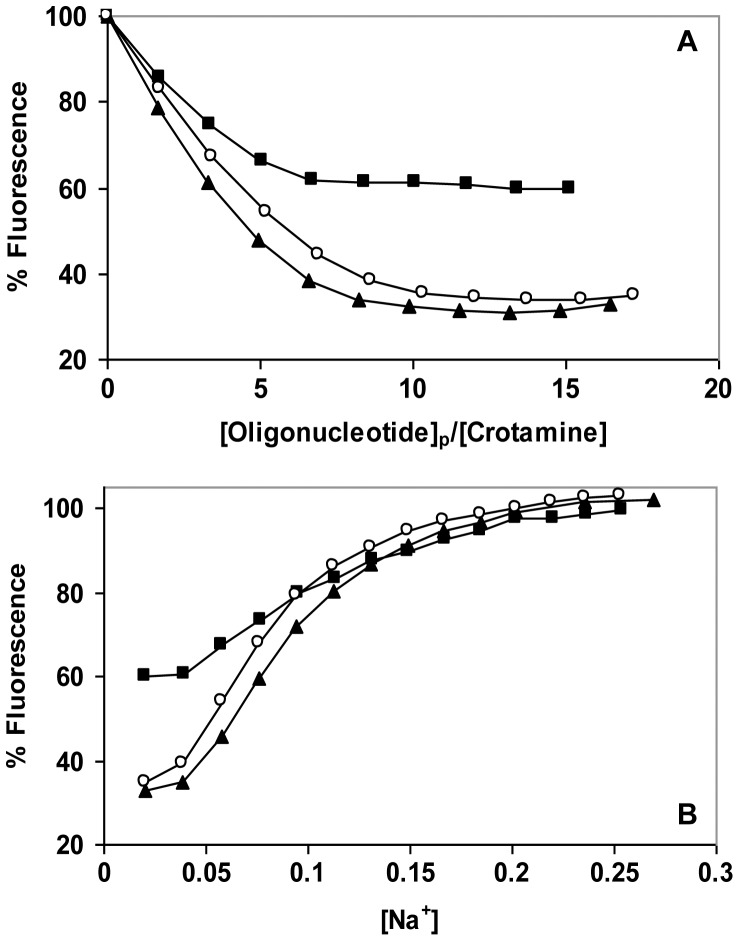
Dependence of binding affinity on oligonucleotide sequence. A. Fluorescence titration in the standard buffer with 0.01 M NaCl: ◯, d(ATGTGGAAAATCTCTAGCAGT) (21+); ▴, d(ACTGCTAGAGATTTTCCACAT) (21-); ▪, duplex (21+/−). B. NaCl-induced reversal.

With this in mind, we examined the binding of an A-T rich 21-residue oligomer, d(ATGTGGAAAATCTCTAGCAGT), its complement, and its duplex with its complement. As seen in Fig. 6A, the maximal extent of crotamine Trp fluorescence quenching achieved with both ss oligos is ∼65%, similar to the fluorescence change seen with d(CCG)_8_C. This is significantly greater than the quenching seen with the A-T rich duplex, ∼40%. Thus, analogous to d(CCG)_8_C and its duplex with its complement, there is a potential for greater quenching with single strands, raising the possibility that one or both of the protein’s Trp residues are involved in this interaction (see below). The salt-back data for the A-T rich oligos (Fig. 6B) show 50% reversals at 0.08, 0.09, and 0.095 M Na^+^ for, respectively, d(ATGTGGAAAATCTCTAGCAGT), its complement, and the duplex. These results are similar to the salt-reversal seen with d(CCG)_8_C, but unlike the variations among the G-C substrates, both A-T rich single strands and their duplex show essentially the same dependence on [Na^+^]. Thus, although there is no general preference for single- *vs.* double-strands, DNA sequence, composition, and structure, it seems that they all can affect the affinity for crotamine.

**Figure 7:**
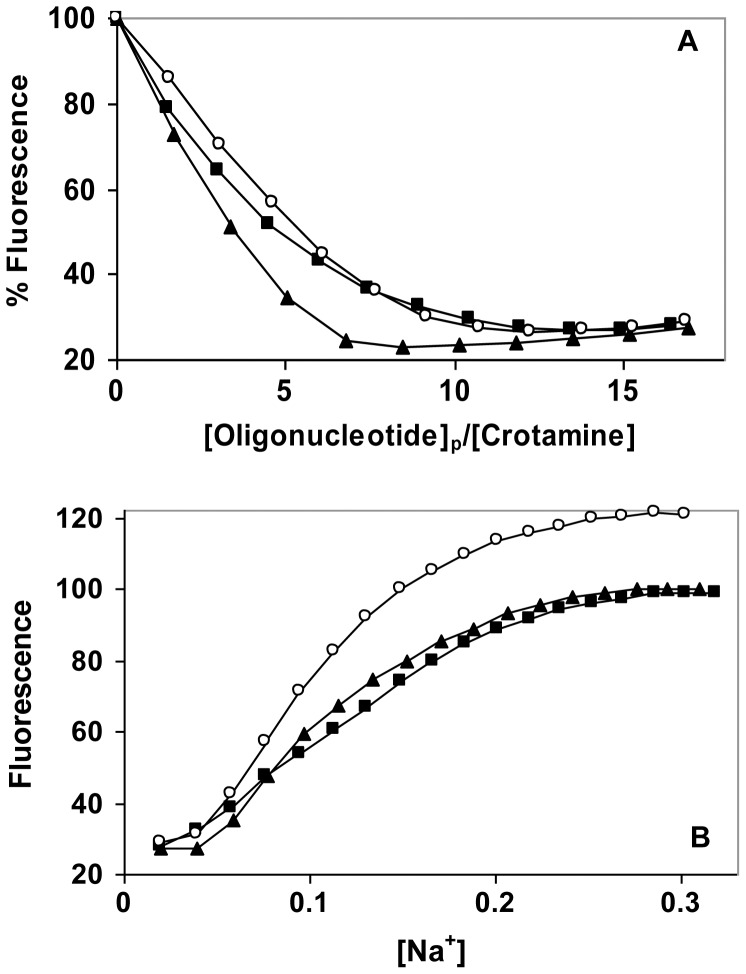
Effect of oligonucleotide length on binding affinity. A. Fluorescence titration in the standard buffer with 0.01 M NaCl: ▪, dT_7_; ▴, dT_14_, ◯, dT_21._ B. NaCl-induced reversal.

The effect of oligonucleotide length on crotamine binding was also examined. In these experiments, oligothymidylates containing 7, 14, and 21 residues were compared (Fig. 7). The three oligonucleotides showed similar titrations, with the 50% reversal points at 0.12, 0.11, and 0.10 M Na^+^ for, respectively, dT_7_, dT_14_, and dT_21_. Here again, there is no obvious preference for length within the ±0.01 M uncertainty in [Na^+^] at 50% reversal. The result is fully consistent with the non-cooperative binding displayed by the quantitative analysis of the d(CCG)_8_C binding data, since with a cooperative interaction the overall affinity would be significantly boosted with oligonucleotides capable of binding an increasing number of crotamines. Given that the salt-dependence is seen to be essentially independent of oligo length, this clearly does not occur.

### Modes of Interaction with DNA

**Figure 8:**
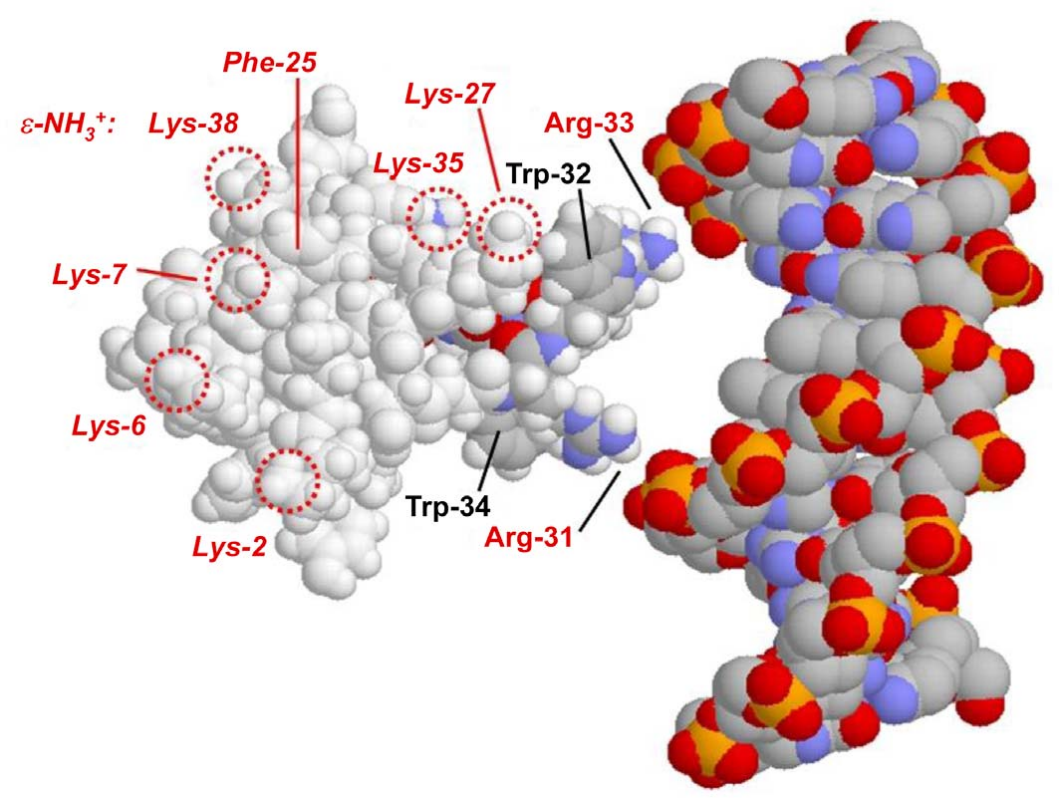
Possible model for crotamine – double stranded DNA major groove interaction. Crotamine (1h50.pdb) and DNA (1bna.pdb) were generated using Rasmol. [46] Sidechains of potentially interacting arginine and tryptophan residues are indicated. The locations of the six lysine ε-NH_2_ groups visible in the pictured orientation are indicated by circles.

With 11 basic amino acids (9 lysine and 2 arginines) and 5 aromatics (2 tryptophans, 2 phenylalanines, and 1 tyrosine), there are many ways that nucleic acids could conceivably bind to the surface of crotamine. One potential binding site is the region between residues 31 and 35, Arg-Trp-Arg-Trp-Lys. This combination of alternating basic and aromatic residues is similar to the sequence of short peptides known to possess significant nucleic acid binding affinity, with greater affinity for ss than for ds DNA [40]–[44]. In the case of crotamine, we have not seen such a preference. This could be due to the particular geometry of these residues within the 3D structure of the protein. As seen in Fig. 8, positioning crotamine within the major groove of the dsDNA B-helix facilitates backbone phosphate interactions with Arg-31 and Arg-33. Moreover, Trp-32 is in a position to flip its indole ring into the groove.

**Figure 9:**
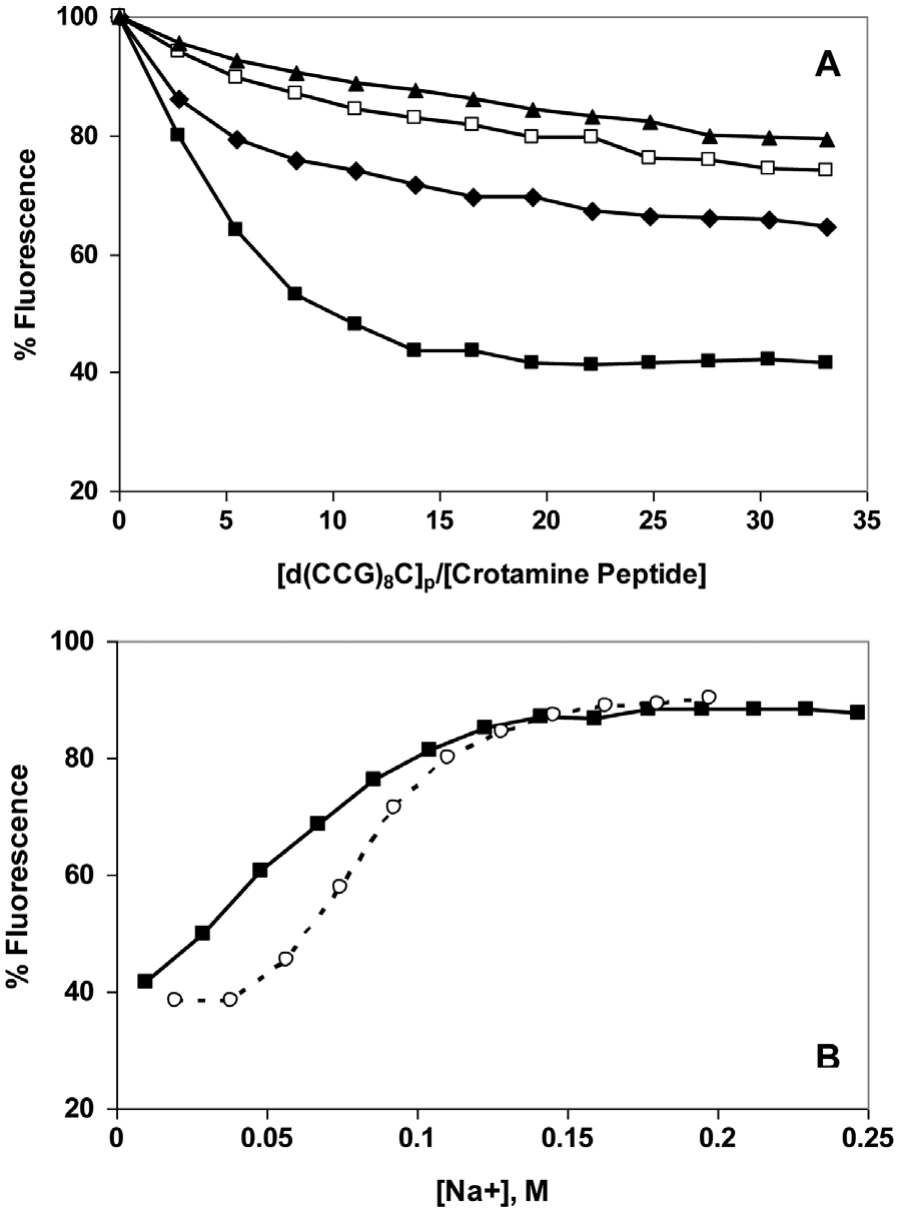
Binding of d(CCG)_8_C to Arg-Trp-Arg-Trp-Lys-Leu-NH_2_ as a function of [NaCl]. A. Fluorescence titrations were performed as in Fig. 3, with the following [NaCl]: ▪, 0.01 M; ♦, 0.05 M; □, 0.075 M; ▴, 0.1 M. B. NaCl reversal: ▪, reversal of 0.01 M titration of Arg-Trp-Arg-Trp-Lys-Leu-NH_2_ with d(CCG)_8_C_;_ ◯, reversal of 0.01 titration of crotamine with d(CCG)_8_C (data from Fig. 3B).

With a view toward establishing the plausibility of this region as a nucleic acid binding surface, we synthesized the hexapeptide, Arg-Trp-Arg-Trp-Lys-Leu-NH_2_, containing the sequence of residues 31–35, and examined its DNA binding properties. At low salt (0.01 M NaCl), the effect of d(CCG)_8_C on the peptide’s tryptophan fluorescence is similar to that seen with the full-length protein (Fig. 9). However, at increasing salt levels, the binding is clearly much weaker than it was for the full-length protein. In 0.05 M NaCl, where the protein was saturated by the oligonucleotide at an [oligo]_p_:[crotamine] of 22∶1, a higher [oligo]_p_:[peptide] (33∶1) achieved less than half saturation – even below that seen for the protein at 0.075 and 0.1 M NaCl (where the peptide shows less than 25% saturation). This difference is also illustrated by the salt-back after titration at 0.01 M NaCl (Fig. 9B). Whereas half-reversal of binding for the full-length protein is achieved at 0.08 M [Na^+^], the short peptide clearly requires less Na^+^ to reach this point (0.06 M). In addition, the reversal seen with the short peptide occurs over a broader range of [Na^+^], indicating a weaker dependence of affinity on [salt]. We also note that in this comparison, NaCl was added at a higher [oligo]_p_:[peptide] (33∶1) than for the full-length protein (22∶1), which would (by mass action) have a greater effect on stabilizing complex formation. Thus, the salt-reversal clearly shows a weaker affinity and salt-dependence for the peptide.

Compared to the full-length protein, the short peptide will lack the larger molecule’s 3D structure and have significantly greater conformational flexibility. This might explain, in part, the difference in affinity and salt-dependence. In addition, as seen in Fig. 8, the intact protein offers other potential binding sites for DNA, which could account for the higher affinity and salt dependence. Six of the nine lysine residues in crotamine are visible in Fig. 8. Two are in close proximity to the arginines, but four (residues 2, 6, 7, and 38) are distal (about 30 Å from the Arg-31 and-33). A second DNA, or a cell-surface heparin/heparan sulfate could conceivably be bound at this second location. An aromatic residue, Phe-25, is in close proximity to these four lysine residues (Fig. 8), and, analogous to the potential role of the tryptophan(s), could contribute to the non-electrostatic component of binding. In this regard, as we noted, at least some of the affinity of crotamine for heparin is the result of non-ionic interactions. [12].

Another consequence of the large number of basic residues (and small number of acidic amino acids) is the tendency of the protein to form aggregates when complexed with DNA. With its multiple potential binding sites, each crotamine is potentially capable of binding at least two DNA molecules, which, if they are of sufficient length, could in turn bind additional crotamines. In this manner, a protein-DNA network would form and grow, eventually leading to aggregation and precipitation. Although there is no direct evidence for this phenomenon, the absence of aggregation with relatively short oligonucleotides is consistent with their inability to simultaneously bind to more than one crotamine.

The ability of crotamine to bind to multiple negatively-charged macromolecular substrates, and in turn form aggregates, is likely to be essential to its functioning as a drug delivery vehicle. The involvement of cell surface heparan sulfate proteoglycans in the uptake of crotamine and crotamine-DNA complexes implies concurrent binding of the DNA and polysaccharide. [12] Moreover, the transfection and cellular uptake efficiencies of small arginine-rich peptides complexed with DNA was found to be a function of the size of the resulting aggregates. [45] Transfection increased with aggregate size, whereas internalization of the DNA was more efficient with small complexes. [45] With this in mind, we are currently investigating the sizes of crotamine-DNA aggregates and the rates at which they form, with a view to determining the optimal particle size and incubation times for DNA delivery into cells.

### Conclusions

We have shown that crotamine, a basic polypeptide component of the venom of the South American rattlesnake, forms aggregates with DNA molecules longer than ∼ 50 bp. Shorter DNA also binds the protein, and we have quantified the affinities and site size of the non-aggregated complexes that form. Complex formation occurs with both double- and single-stranded DNA, is non-cooperative, and there is no obvious dependence of binding parameters on base composition. A detailed analysis of the salt dependence of binding indicates that as many as 3 ionic interactions occur between each crotamine polypeptide and its DNA binding site. With these observations, and inspection of the three-dimensional structure of crotamine, we have identified a potential positively-charged DNA-binding surface on crotamine, residues 31–35, Arg-Trp-Arg-Trp-Lys. A hexapeptide containing this sequence binds DNA similarly to the full-length protein, but with a reduced affinity and salt dependence. This result and the presence of many other basic residues on the protein suggest that crotamine may interact with DNA in a variety of ways, which would also facilitate the formation of aggregates. Given the specificity of crotamine for AP cells, [8], [12], [17], [25] studies of this nature should prove valuable in the development of effective DNA drug delivery vehicles.
